# Effects of Red Mud on Microstructures and Heat Resistance of ZL109 Aluminum Alloy

**DOI:** 10.3390/ma18030664

**Published:** 2025-02-02

**Authors:** Zhuofang Huang, Anmin Li, Wendi Zhou, Jinjin Li, Jinkai Zhang

**Affiliations:** 1 School of Resources, Environment and Materials, Guangxi University, Nanning 530004, China; 2015301031@st.gxu.edu.cn (Z.H.); 2139200239@st.gxu.edu.cn (W.Z.); 2139200216@st.gxu.edu.cn (J.L.); 2139200229@st.gxu.edu.cn (J.Z.); 2 MOE Key Laboratory of New Processing Technology for Nonferrous Metals and Materials, Guangxi University, Nanning 530004, China; 3 State Key Laboratory of Featured Metal Materials and Life-Cycle Safety for Composite Structures, Guangxi University, Nanning 530004, China

**Keywords:** aluminum alloys, red mud, heat-resistant, Al_5_Cu_2_Mg_8_Si_6_, Al_7_Cu_4_Ni, Orowan mechanism

## Abstract

The effects of red mud on the microstructures and high-temperature tensile properties of the ZL109 aluminum alloy have been investigated. Red mud/ZL109-based composite materials with added red mud (a major byproduct of the aluminum industry), which has been coated with nickel by chemical deposition, have been prepared through gravity casting. The results show that the addition of red mud promotes the alloy’s microstructure and helps to uniformly distribute the eutectic silicon. It also increases the content of heat-resistant phases, such as the Q-Al_5_Cu_2_Mg_8_Si_6_ and γ-Al_7_Cu_4_Ni phases. These changes significantly enhance the alloy’s high-temperature tensile properties. The alloy with 1% (wt.%) red mud exhibits the best tensile strength at both room temperature and 350 °C, reaching 295.4 MPa and 143.3 MPa, respectively. The alloy with 1.5% (wt.%) red mud demonstrates excellent performance at 400 °C, achieving a tensile strength of 86.2 MPa through the cut-through method and Orowan mechanism. As a reinforcing material, red mud not only improves the high-temperature resistance of the aluminum alloy but also provides a way to recycle industrial waste. This study offers a new way to address the red mud waste problem and helps develop high-performance, heat-resistant aluminum alloys. It shows the potential of these alloys in high-temperature applications.

## 1. Introduction

Al-Si alloys are widely used in manufacturing lightweight automotive components, such as engine blocks, cylinder heads, and pistons, due to their low density, high strength, and excellent casting performance [1,2,3]. In recent years, as the automotive industry has placed higher demands on aluminum alloy components for high-temperature environments [4], the rapid degradation of Al-Si alloys’ mechanical properties at elevated temperatures has become a key issue limiting their broader development and application. Therefore, it has become essential to develop novel high-strength, heat-resistant cast Al-Si alloys. A common approach to enhancing the mechanical properties of cast Al-Si alloys is the addition of Cu and Mg elements. However, when the temperature exceeds 200 °C, the β-Mg_2_Si and θ-Al_2_Cu phases in the alloys rapidly coarsen, which leads to a decline in material performance. Consequently, the service temperature for this type of alloy is generally limited to below 200 °C [5,6]. To enable Al-Si alloys to be used above 250 °C, Ni is introduced into the cast Al-Si-Cu-Mg alloy, forming a high-strength, heat-resistant Al-Si-Cu-Ni-Mg casting alloy. The addition of Ni promotes the formation of heat-resistant phases such as ε-Al_3_Ni, δ-Al_3_CuNi, and γ-Al_7_Cu_4_Ni [7], which maintain excellent thermal stability even above 300 °C. However, in cast Al-Si-Cu-Ni-Mg alloys, the Si content typically exceeds 11%, nearing the eutectic composition, resulting in the formation of coarse primary Si phases in the alloy [8]. These coarse phases easily induce stress concentration underloading, thus making the alloy’s mechanical properties deteriorate. By adjusting the size and distribution of the coarse Si phases and Al-Cu-Ni phases in cast Al-Si-Cu-Ni-Mg alloys, it is possible to enhance the alloy’s mechanical properties at elevated temperatures, thereby expanding its application potential in high-temperature environments [9,10]. In short, optimizing the composition and the heat treatment procedures can improve the overall performance of cast Al-Si alloys and ensure that they maintain superior mechanical properties under more stringent high-temperature conditions, meeting the complex requirements for outstanding performance materials in modern industry.

Aluminum matrix composites (AMCs) have emerged as novel structural materials due to their excellent properties. In previous studies, the addition of ceramic particles such as SiC, TiB_2_, and TiC have been shown to significantly improve the high-temperature performance of aluminum alloys [11,12,13]. For example, Bian et al. [14] prepared multiphase (Al_2_O_3_ + ZrB_2_ + AlN)/Al particle-reinforced aluminum composites via an in situ method, where AlN was intended to improve heat resistance, while ZrB_2_ and Al_2_O_3_ enhanced material rigidity and thermal stability. Studies have shown that these composites exhibited excellent thermal stability and significantly enhanced high-temperature tensile properties. Additionally, it is noted that the size and distribution of the reinforcement particles, as well as their bonding with the aluminum matrix, are crucial factors for improving high-temperature performance. Shen et al. [15] prepared TiB_2_/Al-Cu-Li composites using a mixed salt reaction of K_2_TiF_6_ and KBF_4_ and found that a large amount of δ’ (Al_3_Li) phase in the composite significantly improved its mechanical properties. Zhao et al. [16] revealed that the tensile strength of TiB_2_/Al-Cu-Li composites increased by 152 MPa with the addition of TiB_2_ particles, attributing this to the presence of abundant T1 (Al_2_CuLi) and S (Al_2_CuMg) phases in the composite.

However, the poor wettability between ceramic particles and the metal melt, such as the 90.5° contact angle between Al_2_O_3_ and the aluminum melt, makes ceramic–metal bonding challenging [17,18]. Ceramics are typically composed of covalent bonds, while metals consist of metallic bonds, making effective chemical reactions between them difficult. Therefore, the bonding strength at the ceramic–metal interface is often weak, and ceramic particles tend to detach during service. To improve the situation, various surface modification techniques have been developed to improve ceramic–metal wettability, such as sol–gel [19,20], ball milling [21], and electroless plating. Hong et al. [22] successfully prepared a uniform nickel coating on the surface of zirconia-toughened alumina (ZTA) ceramics through electroless plating. Similarly, Guo et al. [23] used electroless plating to prepare a Co layer with an average thickness of 50–100 nm on WC particles with a diameter of 0.3–0.5 μm. The results showed that the electroless plating rate was exponentially related to the temperature, and the coating thickness was correlated with the solution’s pH value. These surface modification techniques not only improved wettability but also enhanced the bonding strength between ceramic particles and the metal matrix, thereby improving the overall performance of the composite material.

Red mud, the main solid waste generated during alumina production, appears reddish-brown due to its high iron oxide content. In dry form, red mud has a loose and soft texture, but it becomes muddy and solid when wet. This untreated red mud is composed of up to eight different mineral components and exhibits uneven particle size, high water absorption, and strong alkaline corrosion. Over the past 50 years, no economically viable method has been developed for the large-scale utilization or disposal of red mud. At present, the global stockpile of red mud has exceeded 4.6 billion tons, and it continues to increase at a rate of about 175.5 million tons per year [24,25]. Statistics show that only 15% of red mud is utilized globally [26], with most being handled by piling or landfilling, which reduces land availability and increases environmental pollution. In response to the global challenge of economically managing the accumulated red mud, three main strategies have been developed over the past two decades: reduction [27], resource utilization [28], and extraction [29]. These strategies are subdivided into various technical approaches, particularly in the resource utilization of red mud, which mainly focuses on leveraging its physical and chemical properties to explore its functional applications in different fields, typically by combining red mud with other material matrices to form composites.

Currently, due to its low cost and the ability to manufacture parts with complex geometries, Al_2_O_3_ is widely used as a reinforcement particle in the production of ceramic-reinforced iron-based wear-resistant materials. Considering the quality of coatings, cost factors, and industrial scalability, electroless plating is an ideal surface treatment method and has been successfully applied to Al_2_O_3_ surfaces [30]. Ni as an intermediate layer is an excellent choice, and nickel coating on alumina is an effective way to promote wettability between aluminum and Al_2_O_3_, significantly improving interfacial bonding.

In this study, firstly, a nickel coating on red mud particles was prepared by chemical deposition, with sodium borohydride as the reducing agent. Ni-coated red mud particles were used as precursors for reinforcing aluminum matrix composites, and the morphology of the Ni coating on the red mud surface was observed. Then, a series of red mud/ZL109 aluminum alloys were prepared via gravity casting, and the morphologies, phase composition, element distribution, room temperature/high-temperature instantaneous tensile properties, and fracture microstructures of these red mud/ZL109 aluminum alloys were studied.

## 2. Methods and Procedures

Al-12Si-1Cu-1Ni-1Mg alloys were prepared by the raw materials, including remelted aluminum ingots with an aluminum content of 99.7% and various intermediate alloys, including Al-50Si, Al-50Cu, Al-20Ni, and Al-50Mg.

The red mud used in this experiment was provided by Guangxi Huayin Aluminum Co., Ltd. (Baise, China). The red mud was strongly magnetically separated prior to use to effectively remove the Fe_3_O_4_ component. The primary chemical composition of the red mud is shown in Table 1.

After being dried, the red mud was manually ground for 30 min to refine the particles, then added to a saturated NiSO_4_ solution, and subjected to ultrasonic vibration for 10 min to ensure thorough dispersion. Next, the mixture was stirred in a magnetic stirrer at 200 r/min for 1 h to ensure adequate mixing of the red mud with the solution. After being stirred, the solution was filtered and washed multiple times with deionized water. The filtered red mud was placed in a forced-air drying oven and dried for 3 h to ensure complete dehydration. The dried red mud was ground again for 30 min and then added to a preheated (50 °C) mixed solution of NaBH_4_, NaOH, and trisodium citrate (C_6_H_5_Na_3_O_7_) in a thermostatic water bath (Aladdin, Shanghai, China). It was stirred at 200 r/min for 10 min to conduct the electroless plating process. After electroless plating, the red mud was filtered, thoroughly rinsed with deionized water to remove residual chemicals, and finally dried in an oven to ensure complete dehydration. The specific composition of the solution is shown in Table 2.

The melting process was conducted in a JJZP-45 model medium-frequency induction furnace (JinJi, Shanghai, China). The detailed alloy melting process is as follows: firstly, all intermediate alloys (e.g., Al-Si, Al-Cu, Al-Ni, and Al-Mg) were preheated in a muffle furnace at 250 °C and held for two hours; secondly, pure aluminum ingots were remelted at 700 °C. When the thermocouple thermometer (ChengXin Instrument, ShenZhen, China) indicated that the furnace temperature had reached 720 °C, the preheated Al-Si intermediate alloy was added. After holding for 10 min, the melt was thoroughly stirred to ensure uniformity. When the melt temperature reached 730 °C, the preheated Al-Cu and Al-Ni intermediate alloys were added in sequence, followed by holding and stirring to ensure complete dissolution and uniform distribution of the intermediate alloys. The melt was then cooled to 700 °C, and the Al-Mg intermediate alloy wrapped in aluminum foil was added and pressed below the melt surface with a skimmer to ensure rapid dissolution and uniform mixing. Once all alloys were fully melted, mechanical stirring was performed using a graphite stirrer at a rate of 600 r/min for approximately 10 min. During this process, the preheated red mud was gradually added to ensure its uniform dispersion in the melt. After all the red mud was added, the melt was heated to 720 °C, and 1% (wt.%) of C_2_Cl_6_ was added as a refining agent for degassing and slag removal. During refining, a skimmer was used to remove floating slag from the melt surface to ensure the purity of the metal melt. After the refining was completed, the melt was left for 10 min to allow the residual gases and impurities in the melt to emerge fully, and then the melt was cast at 700 ° C into a metal mold preheated at 200 ° C.

The ingot was heat-treated as follows: firstly, the ingot was placed in a muffle furnace, heated to 515 °C, and held for 8 h to complete the solution treatment; secondly, the ingot was quickly quenched in water at 90–100 °C to retain the supersaturated solid solution structure formed at a high-temperature solution state, thus creating conditions for aging strengthening to the greatest extent. Finally, after quenching, artificial aging treatment was conducted. The ingot was reheated in a muffle furnace to 175 °C and held for 8 h. After the heat preservation was completed, the ingot was cooled with the furnace to control the cooling rate, so that the precipitated phase could have stable distribution, and avoided the internal stress or uneven phase precipitation caused by too-fast cooling.

For analysis, samples were taken from the central portion of the bottom of the casting. The sample surface was sequentially polished, first using sandpapers of 400#, 600#, 800#, 1200#, and 2000# grits for preliminary treatment, and then polished on a P-2G metallographic polishing machine (JingBo Instrument, ShaoXing, China) to ensure a smooth, flat surface. The samples were then cleaned with ethanol in an ultrasonic cleaner (Sunne Instrument, ShaoXing, China)and etched with Keller’s reagent for 16 s to reveal the microstructure. After etching, the samples were immediately rinsed with water and sprayed with alcohol to expedite drying. To confirm the chemical composition of the alloys, an ISPARK 8860 Optical Emission Spectrometer (OES) from ThermoFisher(Massachusetts, USA) was used. To characterize the crystal structure, X-ray diffraction (XRD) analysis was conducted using a Rigaku D/MAX 2500V XRD analyzer (Rigaku, Tokyo, Japan) with a Cu target. The experimental voltage was set to 40 kV, with a testing angle range of 5° to 90° and a scanning speed of 5°/min. Additionally, various scanning electron microscopes (SEMs) and energy-dispersive spectrometers (EDSs) were used for microstructure observation, including the Hitachi S-3400N, SU8020 (Hitachi, Tokyo, Japan), and the Zeiss Sigma 300 (Zeiss, Shanghai, China). Detailed microstructure including phases and compositional analyses were performed using a FEI Tecnai G2 F20 transmission electron microscope (TEM) equipped with EDS (FEI Tecnai, Shanghai, China).

For mechanical performance testing, room temperature tensile tests were conducted using an AGS-X 100 KN universal testing machine (SHIMADZU, Shanghai, China) to evaluate the tensile properties of the material. For high temperature mechanical performance testing, a ZWICK/Roell Kappa 100 KN creep testing machine(ZWICK/Roell, Ulm, German) was used. After heating to the target temperature, the sample was held for 20 min to ensure a uniform and stable temperature before conducting the tensile test. Each test was repeated three times to ensure the reliability and reproducibility of the results.

## 3. Results and Discussions

### 3.1. Characterization of Ni-Coated Red Mud Particles

**Figure 1:**
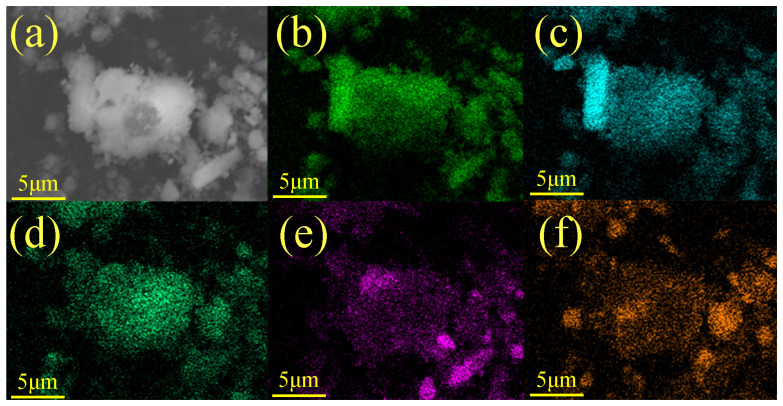
EDS surface scan results of Ni-coated red mud particles: (**a**) SEM figure of red mud, (**b**) O, (**c**) Al, (**d**) Ni, (**e**) Ca, and (**f**) Fe.

The SEM images of red mud@Ni particles coated via ion-assisted electroless plating are shown in Figure 1. The chemical compositions of the ZL109 alloy are shown in Table 3. EDS characterization of the red mud particles reveals a Ni coating layer on the surface, with a Ni content between 5% and 10% as shown in Table 4. This level of Ni coating is sufficient to promote interfacial reactions and improve interfacial wettability in the subsequent melting process.

### 3.2. Microstructure Analysis and Discussion of Red Mud/ZL109 Alloy

**Figure 2:**
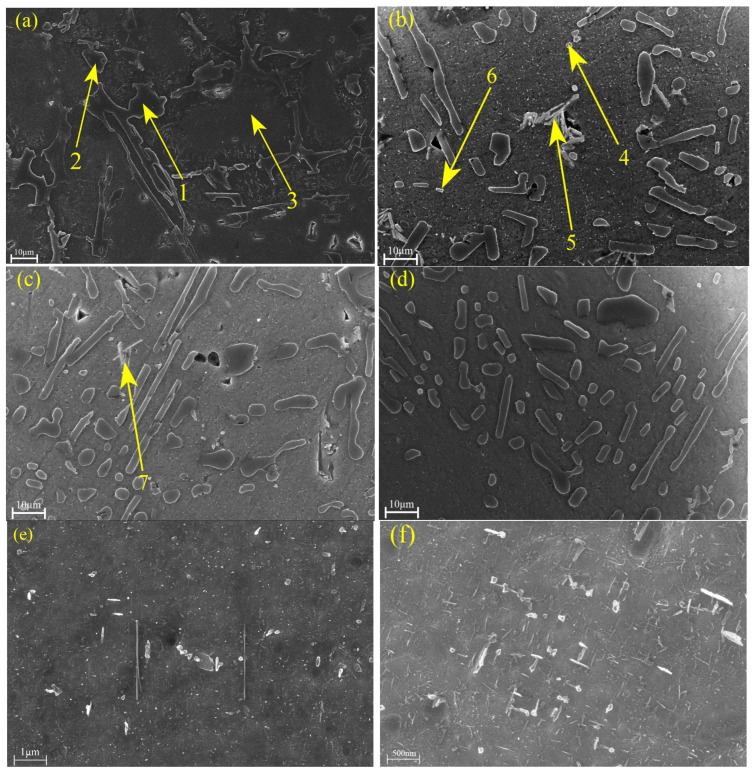
SEM images of red mud/ZL109 alloys with red mud content (wt.%): (**a**) 0%, as-cast; (**b**) 1%, T6; (**c**) 1.5%, T6; (**d**) 2%, T6; (**e**) 1%, T6; and (**f**) 1.5%, T6.

The typical microstructures of T6-treated red mud/ZL109 alloys with varying amounts of red mud additions are shown in Figure 2a–d. As seen in Figure 2, all four types of T6-treated red mud/ZL109 alloys contain α-Al dendrites, Si particles of various morphologies, and intermetallic phases.

By combining EDS and XRD analysis, phase identification of the alloy’s composition is achieved, as shown in Table 5. Eight distinct phases are identified [31,32,33]. In Figure 2a, the primary Si phase (labeled 1) appears as large, black blocky structures, while the eutectic Si phase (labeled 2) is displayed as black, lamellar structures. In Figure 2b, the θ’-Al_2_Cu phase (labeled 4) appears as white, spherical structures. The Q phase (labeled 5), with lattice parameters of c = 0.405 nm and a = 1.04 nm and containing 21 atoms per unit cell, has been referenced in some studies as Al_5_Cu_2_Mg_8_Si_6_ [34,35,36,37]. The δ-Al_3_CuNi phase (labeled 6) appears gray-white after etching and resembles the γ-Al_7_Cu_4_Ni phase, which appears as a white, skeletal structure in Figure 2c (labeled 7). As shown in Figure 2, significant changes occur in the alloy’s microstructure after T6 heat treatment. The eutectic Si transforms into finer needle-like or spherical structures, optimizing the balance between strength and ductility. In contrast, the morphologies of some Al-Ni intermetallic compounds remain largely unchanged. During the process of heat treatment, part of the Cu element dissolves into the matrix, which not only makes the size of the Al-Cu-Ni phase become small, but also part of the skeletal structures becomes fragmented. These changes in Al-Cu-Ni phases reduce the cleavage of the second phase on the matrix and also the stress concentration, in which the closed or semi-closed Al-Cu-Ni phases were essential strengthening phases during high-temperature deformation [38].

**Figure 3:**
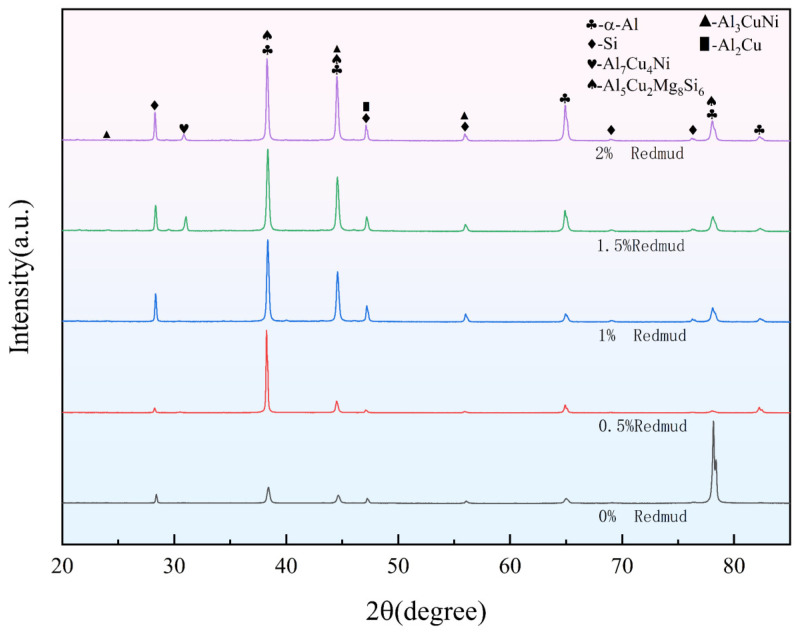
XRD patterns of alloys with various red mud contents.

Figure 3 shows the XRD patterns of alloys with various red mud contents. From these results, it can be observed that, after adding red mud, the peak intensities of the reinforcing phases increase correspondingly. Notably, the γ-Al_7_Cu_4_Ni phase reaches its highest peak intensity with a 1.5% red mud addition, while the Q phase shows its maximum peak intensity after adding 1% red mud. This is also supported by the microstructure observed in Figure 1, where the addition of red mud correspondingly increases the volume fraction of reinforcing phases (i.e., γ-Al_7_Cu_4_Ni phase and Q phase) in the alloy and alters the intensity of the Al (311) peak.

**Figure 4:**
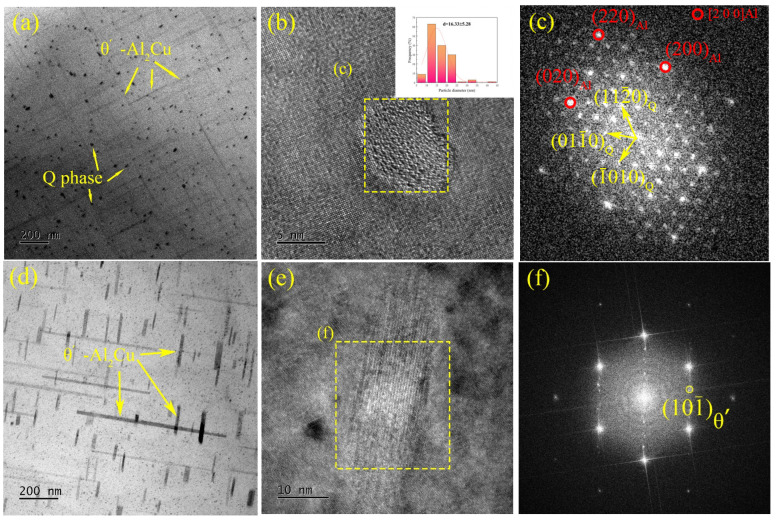

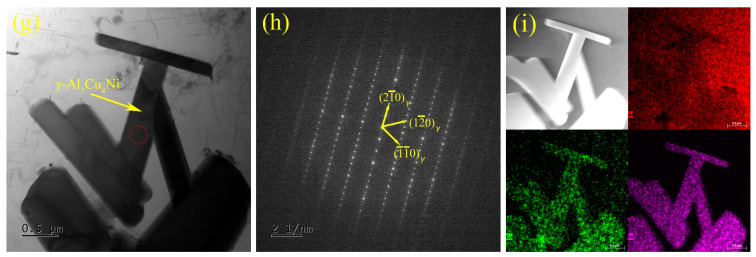
(**a**–**c**) Bright field image, HRTEM and FFT of Q phase; (**d**–**f**) bright field image, HRTEM and FFT of θ’ phase; and (**g**–**i**) bright field image, FFT and EDS results of γ phase.

Through the analysis of the images, diffraction spots, and selected area electron diffraction (SAED) patterns of high-resolution transmission electron microscopy (HRTEM), combined with previous scanning electron microscopy (SEM) images and X-ray diffraction (XRD) analysis, the primary strengthening phases in the T6-treated alloy are identified as the Q phase (Al_5_Cu_2_Mg_8_Si_6_), θ’-Al_2_Cu phase, and γ phase (Al_7_Cu_4_Ni). As shown in Figure 4b, the HRTEM image of the Q phase reveals its characteristic microstructure, while Figure 4c presents the fast Fourier transform (FFT) image of the region marked in Figure 4b. The Q phase primarily appears as elliptical and short rod-shaped forms within the alloy. Analysis of the FFT image in Figure 4c indicates the orientation relationship between the Q phase and the α-Al matrix as (0001)Q // (001)Al and $\left[11\bar{2}0\right]$Q // [110]Al [39]. The elliptical and short rod-like Q phases are evenly distributed in the alloy, which effectively hinders the slip of the dislocation; thus, they will improve the overall mechanical properties of the material.

In addition to two kinds of magnesium-containing nanophases, a needle-like θ’-Al_2_Cu phase with a thickness of less than 5 nm was also observed. The θ’ phase, a common strengthening phase in copper-containing aluminum alloys, exhibits a tetragonal crystal structure (a = 0.404 nm, c = 0.58 nm) and maintains a semi-coherent relationship with the aluminum matrix. This semi-coherent interface will play a key role in strengthening by effectively hindering dislocation motion, thereby enhancing the mechanical properties of the alloy.

Additionally, larger blocky phases are observed, which, as indicated by Figure 4g–i, can be identified as the γ-Al_7_Cu_4_Ni phase. The γ phase exhibits high thermal stability and forms a continuous phase network, which contributes to maintaining material strength at elevated temperatures. Along with other nickel-rich phases, such as the δ phase, it forms a closed or semi-closed network structure, significantly enhancing the load-bearing capacity of the aluminum alloy in high-temperature environments [40].

### 3.3. Tensile Property Analysis

As the temperature increases, both the ultimate tensile strength and yield strength of the materials show a decreasing trend, while the elongation after fracture exhibits an increasing trend. This is because dislocation movement becomes easier at higher temperatures, with thermally activated cross-slip mechanisms becoming dominant, leading to a decrease in material strength and softening of the matrix, thereby enhancing material ductility [41,42,43,44,45,46,47,48,49,50,51].

**Figure 5:**
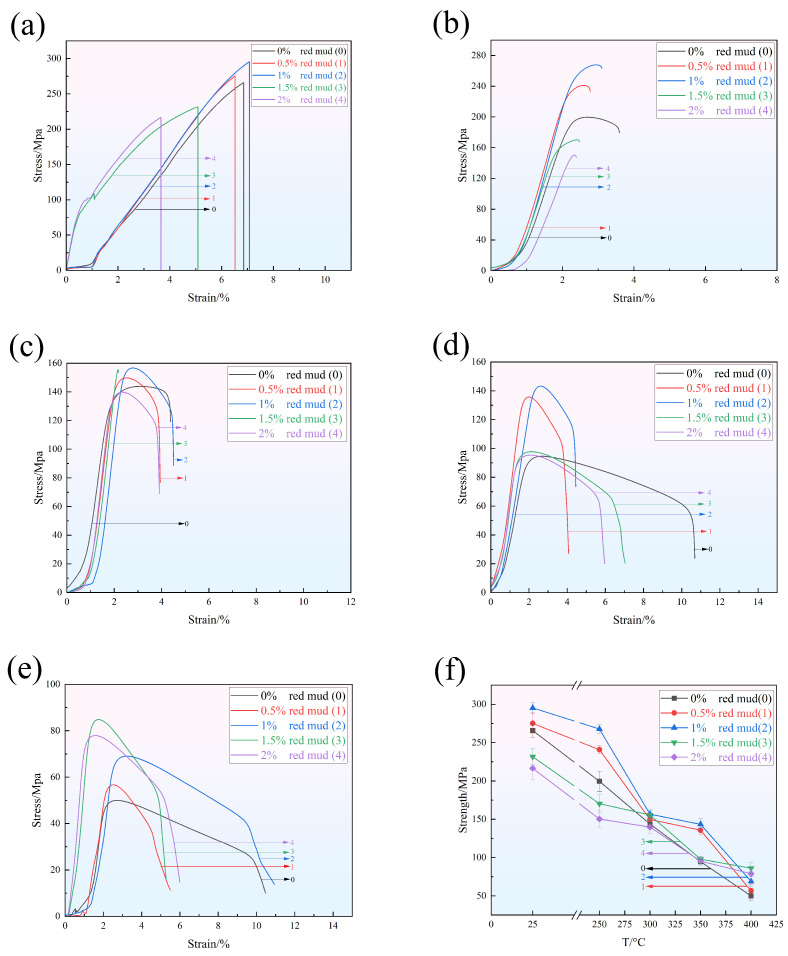
Stress–strain curves of the heat-treated red mud/ZL109 alloys: (**a**) room temperature, (**b**) 250 °C, (**c**) 300 °C, (**d**) 350 °C, (**e**) 400 °C, and (**f**) tensile strength of alloys with various red mud contents at different temperatures.

**Figure 6:**
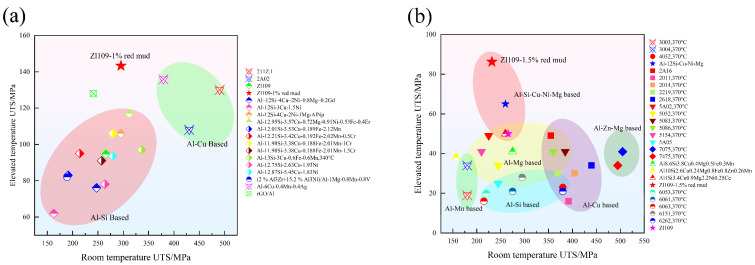
Comparisons between the UTS of the room temperature and elevated temperature of aluminum alloys: (**a**) 350 °C ([41], Total Materia); and (**b**) 400 °C ([42,43,44,45,46,47,48,49,50], Total Materia).

Figure 5 and Table 5 present the instantaneous tensile properties of alloys with different red mud mass fractions (0%, 0.5%, 1%, 1.5%, 2%) tested at 25 °C, 250 °C, 300 °C, 350 °C, and 400 °C, respectively. Figure 6 compares various aluminum alloys at room temperature, 350 °C, and 400 °C. Due to limited data for the tensile test at 400 °C, experimental data for commercial aluminum alloys at 370 °C were obtained from the Total Materia database. As shown in the figures, conventional Al-Si alloys have tensile strengths below 120 MPa at 350 °C and below 30 MPa at 400 °C. In contrast, the alloys in this study demonstrate a strength increase of nearly 20 to 80% at the same temperatures, showing a significant improvement over similar alloys and even double that of some commercial Al-Cu alloys at 400 °C.

At 25 °C, the room-temperature tensile properties of these series of alloys initially increase with red mud content and then decrease. When the red mud content is 1%, the tensile strength and elongation after fracture reach 295.4 MPa and 7.08%, respectively, representing increases of 11.1% and 3% compared to the alloy without red mud. However, when the red mud content exceeds 1%, the tensile properties of the alloys start to decline. With further increases in red mud content, however, agglomeration occurs during melting, negatively impacting the alloy’s room-temperature tensile properties.

At 250 °C, the alloy with 1% red mud content exhibits the best tensile properties, with the tensile strength and elongation after fracture of 267.8 MPa and 3.11%, respectively, indicating that an appropriate amount of red mud at this temperature is helpful to improve both strength and ductility. When the temperature is raised to 300 °C, the tensile strength and elongation after fracture of the 1% red mud alloy increase by 9% and 3.2%, reaching 156.7 MPa and 4.51%. At 350 °C, the tensile strength and elongation after fracture of the 1% red mud alloy are 143.3 MPa and 5.95%, respectively, showing a significant improvement over the alloy without red mud. This performance enhancement is attributed to the addition of red mud, which promotes the precipitation of the Q-Al_5_Cu_2_Mg_8_Si_6_ phase. The Q phase maintains good morphology and size at higher temperatures (357 °C), thereby effectively impeding dislocation motion and improving creep resistance of the alloy [51].

When the temperature is further increased to 400 °C, the alloy with 1.5% red mud content demonstrates the highest high-temperature instantaneous tensile performance, with a tensile strength of 86.2 MPa, a 72% improvement over that of the alloy without red mud. This performance enhancement is mainly due to the red mud addition effectively refining the δ-Al_3_CuNi phase, transforming its morphology from skeletal to granular and short-rod shapes, which contributes to improved high-temperature strength [52]. Analysis of Figure 5f shows that the tensile strength of all alloys decreases between 300 °C and 400 °C, but those alloys with 0.5–1% red mud content exhibit a slower strength decline between 300 °C and 350 °C, indicating that an appropriate amount of red mud helps maintain strength in the intermediate temperature range. However, when the red mud content exceeds 1%, the strength decreases more rapidly. In the high-temperature range from 350 °C to 400 °C, the alloy with 1.5% red mud content demonstrates better high-temperature stability. Additionally, the addition of red mud promotes the formation of the γ-Al_7_Cu_4_Ni phase, which exhibits high thermal stability at temperatures from 350 °C to 450 °C [53], effectively maintaining the material’s high-temperature strength. This is also evidenced by the prominent γ-phase diffraction peak in the XRD pattern of the alloy with 1.5% red mud. When the red mud content exceeds 1.5%, the tensile properties of the alloy begin to decline, likely due to defects or brittle phases introduced by excessive red mud during melting, which weakens the overall performance.

It is well known that the mechanical properties of cast Al-Si alloys largely depend on their intermetallic characteristics (i.e., size, morphology, and distribution) [54]. The addition of red mud significantly alters the alloy’s microstructures, promoting the formation and refinement of intermetallic compounds, as shown in Figure 2e,f. The Al_3_CuNi and Al_7_Cu_4_Ni phases in the microstructure gradually develop into small blocky morphologies. These hard particles are uniformly distributed in the matrix, effectively reducing stress concentration and allowing stress to be evenly distributed around the particles, thereby significantly enhancing the tensile properties of the alloy, including strength and ductility. However, when the red mud content exceeds 1.5%, the fraction of strengthening phases in the matrix significantly decreases (as shown in the microstructure in Figure 2d and the XRD results in Figure 3), resulting in a reduction in the alloy’s strengthening effect and negatively affecting mechanical properties. This adverse effect may stem from particle agglomeration and excessive consumption of solute elements in the matrix due to the excessive red mud addition, which hinders the precipitation efficiency and uniform distribution of strengthening phases. The intermetallic compounds, such as Al_3_CuNi and Al_7_Cu_4_Ni, can be refined and uniformly distributed with an appropriate amount of red mud addition, forming a “skeleton” structure with good load-bearing capacity, thereby improving the alloy’s tensile strength and ductility. However, with excessive red mud content, intermetallic compounds cannot precipitate uniformly, reducing the material’s overall mechanical performance.

During the casting process, especially when reinforcements are added, small pores may be introduced. These pores negatively affect the mechanical properties of the alloys, especially on strength and ductility. The addition of red mud particles may lead to localized areas of bubbles or unfilled voids, increasing the porosity of the alloy and thus reducing its density and tensile strength. In particular, the mechanical properties of the composites were significantly reduced after the addition of more than 1.5%.

**Figure 7:**
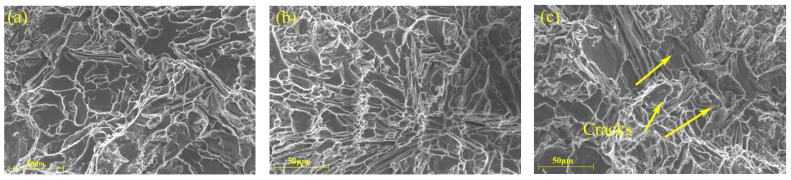

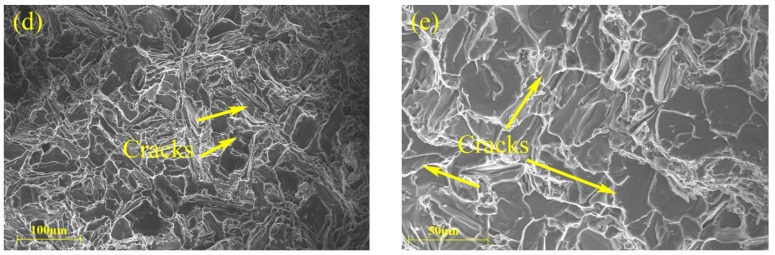
Fracture SEM micrographs of the tensile specimens with different red mud contents (25 °C): (**a**) 0%, (**b**) 0.5%, (**c**) 1%, (**d**) 1.5%, and (**e**) 2%.

**Figure 8:**
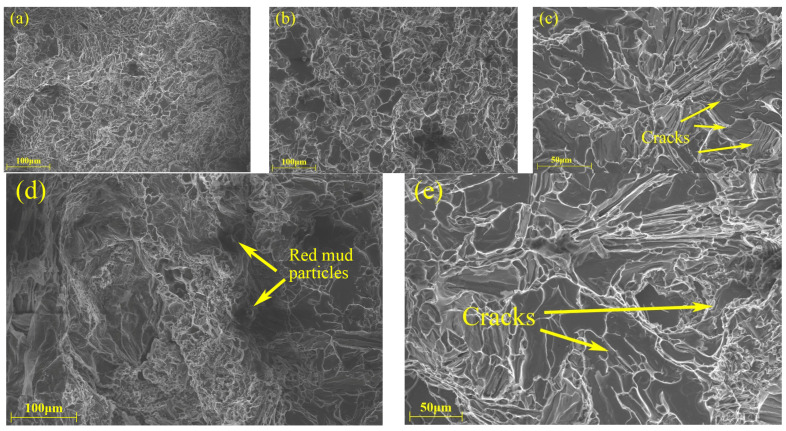
Fracture SEM micrographs of the tensile specimens with different red mud contents (250 °C): (**a**) 0%, (**b**) 0.5%, (**c**) 1%, (**d**) 1.5%, and (**e**) 2%.

**Figure 9:**
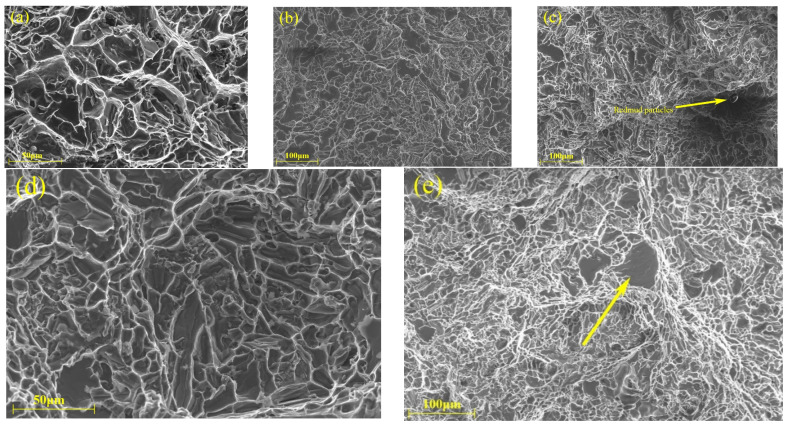
Fracture SEM micrographs of the tensile specimens with different red mud contents (300 °C): (**a**) 0%, (**b**) 0.5%, (**c**) 1%, (**d**) 1.5%, and (**e**) 2%.

**Figure 10:**
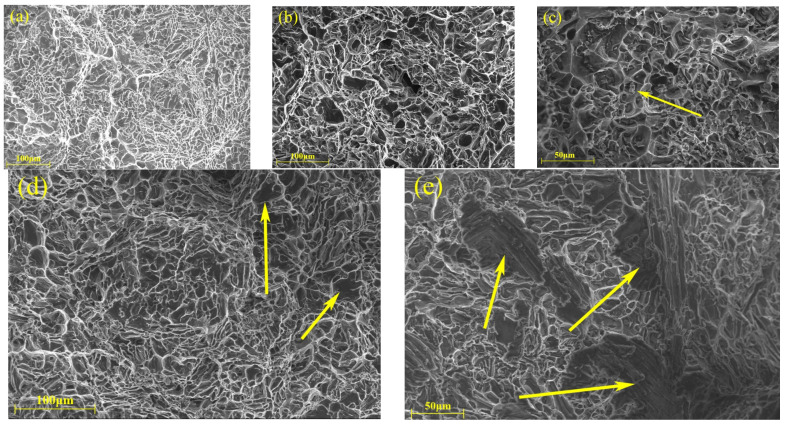
Fracture SEM micrographs of the tensile specimens with different red mud contents (350 °C): (**a**) 0%, (**b**) 0.5%, (**c**) 1%, (**d**) 1.5%, and (**e**) 2%.

**Figure 11:**
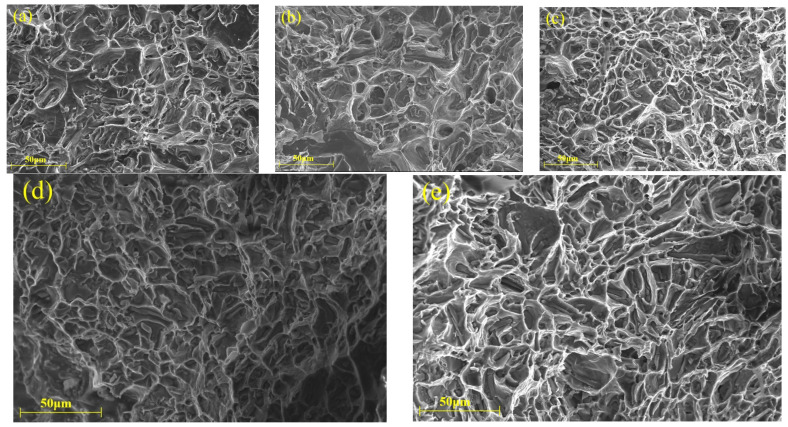
Fracture SEM micrographs of the tensile specimens with different red mud contents (400 °C): (**a**) 0%, (**b**) 0.5%, (**c**) 1%, (**d**) 1.5%, and (**e**) 2%.

To better understand the failure mechanisms of the T6-treated samples at room and elevated temperatures, the fracture surfaces of each of samples with varying red mud contents have been analyzed by SEM. Figure 7, Figure 8, Figure 9, Figure 10 and Figure 11 show the fracture morphologies of tensile specimens tested at 25 °C, 250 °C, 300 °C, 350 °C, and 400 °C. Below 250 °C, the fracture mode of alloys with five different red mud contents primarily exhibits quasi-cleavage fracture. The fracture characteristics include brittle fracture and flat needle-like eutectic silicon fracture surfaces. In the ZL109 alloy with a 1% red mud addition, cracks appear on the cleavage planes, and flaky Si particles and intermetallic compounds (such as Al_3_CuNi) around the matrix cause stress concentration, providing a convenient path for fracture propagation, thereby increasing the likelihood of brittle crack extension.

When the testing temperature exceeds 200 °C, the fracture surfaces of alloys without red mud and with 1.5% red mud display the sign of transition from brittleness to ductility, as shown in Figure 9a,d. This indicates that, as the temperature rises, the failure mode of the material gradually shifts from brittle to ductile fracture. At 300 °C and 350 °C, the fractures primarily exhibit ductile transgranular characteristics, with a mixed brittle-ductile fracture mechanism. In the alloy with 2% red mud, deep gray cleavage planes appear on the fracture surface, indicating some brittle features under elevated temperature conditions. These results of analysis align with previous tensile performance results, confirming that more red mud increases the brittleness of the alloy at 350 °C.

As shown in Figure 11, at 400 °C, all alloys exhibit a ductile fracture mode, with dimples containing multiple fragmented intermetallic compounds. Additionally, as the red mud content increases, the size of the dimples decreases, especially in alloys with 1% and 1.5% red mud. This also explains why these alloys with added red mud significantly enhance the tensile strength at 350 °C and 400 °C. However, when the red mud content increases to 2%, red mud particles are detected at multiple locations on the fracture surface, potentially serving as crack initiation points, which accelerated alloy fractures and led to deteriorated tensile performance.

For eutectic Al-Si-Cu-Ni-Mg alloys, the typical aging precipitation sequence is as follows: θ” (Al_3_Cu) + S” (metastable Al_5_Cu_2_Mg_8_Si_6_) → θ’ (Al_2_Cu) + S’ (Al_5_Cu_2_Mg_8_Si_6_) → θ (stable Al_2_Cu) + Q-Al_5_Cu_2_Mg_8_Si_6_ [55].

As the aging time increases, incoherent θ’, Q, and γ phases precipitate within the matrix, significantly enhancing the strength of the base alloy. The addition of Si, Mg, and Ni elements further enriches the variety of precipitates, forming nano-sized phases containing Si, Mg, or Ni during the aging process. The precipitation of these nano-sized secondary phases is the primary strengthening mechanism for T6-treated alloys. During this process, the strengthening mechanism varies depending on the size of the secondary phase particles. Studies have shown that particles smaller than 20 nm (such as the Q phase) impede dislocation movement through a cut-through method, thereby significantly increasing the material’s strength [56].

In this strengthening mechanism, the additional shear stress (Δ*τ*) generated by nano-phases on dislocations can be calculated using the following formula [57]:

(1) $$\Delta \tau =\sqrt{\frac{3\pi }{2}}\times \frac{\gamma \times \sqrt{f}}{r}$$

Here, *γ* represents the interfacial energy between the two phases, with a value of approximately 0.52 J/m^2^ [37]; *f* is the volume fraction of the secondary phase, around 1.2%; and *r* is the average radius of the secondary phase, approximately 16.33 nm. Based on these parameters, the additional shear stress (Δ*τ*) exerted by the Q phase on the alloy matrix can be calculated to be approximately 7.57 MPa. This means that, under uniaxial tensile conditions, the precipitation of the Q phase could increase the alloy’s yield strength by approximately 10.67 MPa. The refinement effect of these precipitated phases, as well as their interaction with the matrix, plays a significant role in enhancing the alloy’s strength. Small secondary phase particles are precipitated uniformly at the early stage of aging, which, as high-density and evenly distributed strengthening phases, can significantly enhance the ability to resist dislocation slip.

The dislocations cut through these nanoparticles, which increases local shear stress, further improving the alloy’s tensile strength. However, the Q phase coarsens significantly at 400 °C because this temperature exceeds the previously mentioned stability temperature of the Q phase, which results in reducing its strengthening effect.

If the secondary phase particles are larger (e.g., the γ phase), dislocations typically bypass these particles to strengthen the alloy via the Orowan mechanism [58]. The additional shear stress Δ*τ* from this strengthening mechanism can be calculated using the following formula [57]:

(2) $$\Delta \tau =k\times \frac{\mu_{k}\times b}{2\pi \left(l-2r\right)}\times \ln\frac{l-2r}{r_{0}}$$

Here, *k* is a statistical factor, taken as 0.5; *μ_K_* is the average shear modulus, approximately 2.6 − 4.3 × 10^10^ Pa; *b* is the Burgers vector, valued at 0.24 nm; *l* is the average distance between particle centers, approximately 0.75 µm; *r* is the particle radius, approximately 0.21 µm; and *r*_0_ is the dislocation core radius, taken as 2*b*. Using these parameters, the additional shear stress Δ*τ* generated by the γ phase on the matrix can be calculated to be approximately 9.25 to 16.34 MPa. Under uniaxial tensile conditions, the corresponding yield strength increment is about 13.04 to 23.04 MPa.

Considering the combined effect of the Q and γ phases, these precipitated phases theoretically increase the yield strength of the alloy with a 1.5% red mud addition at 400 °C by 23.71 to 33.71 MPa, which aligns well with the experimentally measured yield strength increment of approximately 36.4 MPa. This indicates a synergistic effect between the Q and γ phases during strengthening. The Q phase, due to its nano-scale size, increases material strength through an Orowan bypass mechanism, while the γ phase effectively impedes dislocation slip, further enhancing the high-temperature tensile property of the material.

**Figure 12:**
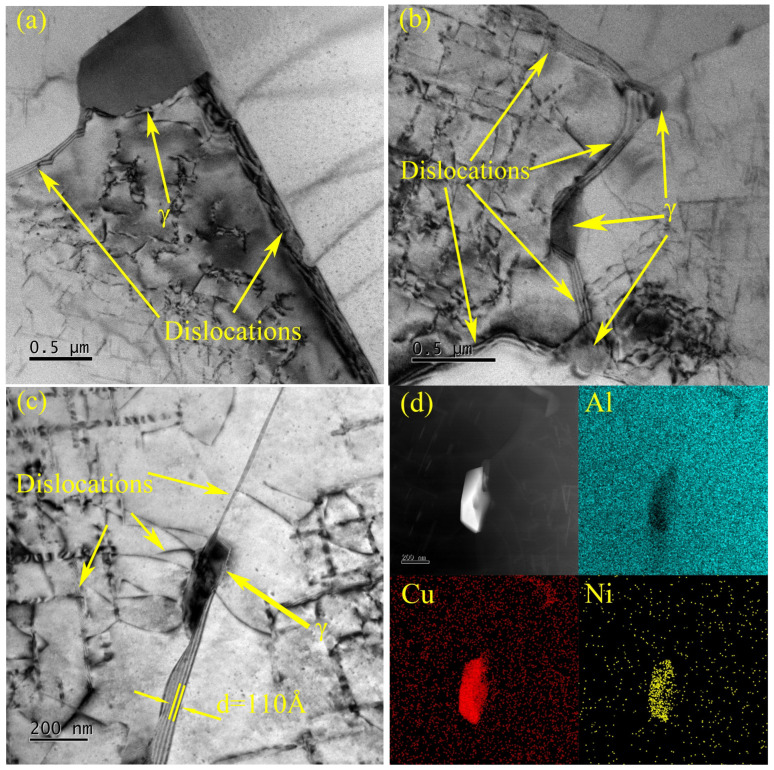
(**a**–**c**): TEM image of reinforced particles γ-Al_7_Cu_4_Ni and dislocations, and (**d**) the EDS result of γ-Al_7_Cu_4_Ni.

As shown in Figure 12, the γ phase, as the primary strengthening secondary phase, maintains structural integrity, especially in high-temperature environments. Next to this secondary phase, the alloy matrix has slippage bands with a width of approximately 110 Å. The thermal stability of the γ phase at elevated temperatures allows it to continue providing effective strengthening under high-temperature conditions, thereby improving the material’s high-temperature tensile strength. This strengthening mechanism explains why the alloy retains exceptional mechanical properties at elevated temperatures [39].

In addition, the morphology of eutectic silicon has a significant impact on the high-temperature mechanical properties of the alloy. The high-hardness eutectic silicon can effectively transfer loads at elevated temperatures. In as-cast alloys, eutectic silicon is typically distributed in a network along grain boundaries, forming a high-strength skeletal structure that mechanically interlocks with the aluminum matrix, thereby mitigating strength degradation in high-temperature environments [59]. However, in T6 heat-treated aluminum alloys, this networked eutectic silicon undergoes spheroidization, causing a partial loss in its ability to transfer high-temperature loads, which may contribute to the observed decrease in high-temperature strength.

Nonetheless, the spheroidized eutectic silicon exhibits enhanced compatibility for coordinated deformation at elevated temperatures, reducing stress concentration caused by networked eutectic silicon and significantly improving the overall mechanical properties of the material. Like the spheroidization of eutectic silicon, high-temperature solution treatment also induces the spheroidization and refinement of secondary phases, such as the AlCuNi phase. This spheroidization and refinement alleviate internal stress concentration, thus reducing the generation of cracks and significantly improving the elevated temperature mechanical properties of the material [39].

The spheroidized eutectic silicon and refined AlCuNi phase exhibit superior stability and deformational compatibility under high-temperature conditions, achieving a balance and enhancement of strength and ductility of the alloy. These phenomena indicate that, by controlling the morphology of eutectic silicon and secondary phases, the alloy’s mechanical performance in high-temperature environments can be significantly optimized, improving its creep resistance and high-temperature strength stability. Especially in T6-treated alloys, the combination of spheroidized eutectic silicon and refined AlCuNi phases achieves a comprehensive optimization of strength and plasticity at elevated temperatures, demonstrating outstanding potential for high-temperature applications.

**Figure 13:**
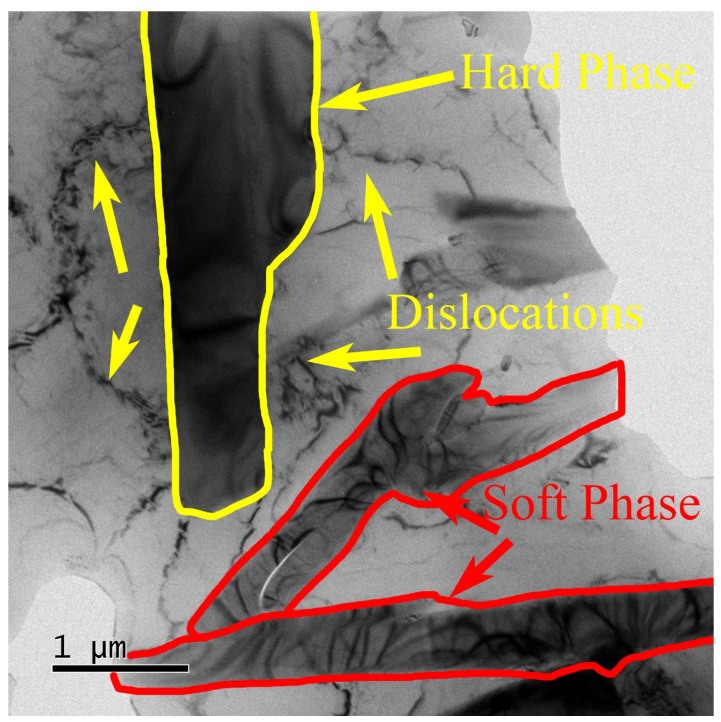
TEM image of the hard phase and soft phase in ZL109/red mud alloys.

As shown in Figure 13, the interaction between hard and soft phases in aluminum alloys enhances the overall mechanical performance of the alloy under high-temperature and high-stress conditions. The hard phases provide high strength, while the soft phases improve toughness and ductility, and their synergistic reinforcement significantly elevates the alloy’s performance. Hard phases (e.g., θ’ phase, Q phase, γ phase) precipitate as fine particles during the aging process, acting as pinning agents that hinder dislocation movement, thereby increasing material strength. The finer and more uniformly distributed the hard phase particles are, the more they contribute to the strength of the material. Additionally, the amount of precipitated hard phases correlates with the content of elements such as Cu and Mg in the alloy; increased Cu content promotes the precipitation of θ’ and Q phases, thereby enhancing yield strength and hardness [60].

However, the presence of hard phases can introduce brittleness and stress concentration, reducing the material’s plasticity. Here, soft phases (such as the aluminum matrix and phases formed with solute elements) absorb stress through plastic deformation, compensating for the brittleness introduced by hard phases, alleviating stress concentration and preventing the complete blockage of dislocation slip, thus improving toughness and ductility. The soft phase provides relatively easy pathways for dislocation movement, which is especially important under high-load conditions, preventing brittle fractures and allowing the material to exhibit enhanced plasticity in high-temperature and high-stress environments.

## 4. Conclusions

1. The addition of red mud improves the microstructure of the ZL109 alloy. It refines and evenly distributes the eutectic silicon. It also increases the content of heat-resistant phases, such as the Q-Al_5_Cu_2_Mg_8_Si_6_ phase and the γ-Al_7_Cu_4_Ni phase. As a result, the alloy’s tensile strength and ductility are greatly improved.
2. With a red mud content of 1%, the alloy exhibits optimal mechanical properties at 25 °C and 350 °C, with tensile strengths of 295.4 MPa and 143.3 MPa, respectively.
3. At the elevated temperature of 400 °C, the alloy with 1.5% red mud demonstrates the highest tensile strength of 86.2 MPa, indicating that an appropriate amount of red mud can effectively improve the alloy’s high-temperature stability and strength through the cut-through method and Orowan mechanism.

This study successfully applied red mud to ZL109 aluminum-based alloys, providing a novel approach to addressing the issue of red mud waste accumulation and paving the way for developing high-performance, heat-resistant aluminum alloys, showing the application potential of this alloy in the field of elevated temperature. In further research, the addition amount of red mud and the processing techniques employed can be further optimized to explore the combined effects of different red mud mass fractions and processing methods on alloy properties. The objective is to identify the optimal red mud content and coating treatment for achieving the best mechanical properties. The interfacial interaction between red mud and the aluminum alloy matrix remains a key issue. Subsequent studies will investigate the bonding mechanism between red mud particles and the alloy matrix, with a focus on its stability and durability in high-temperature environments. Furthermore, research could be expanded to investigate the enhancement effects of red mud in various alloy systems, with a particular focus on other common high-temperature aluminum alloys (e.g., aluminum–silicon alloys, aluminum–copper alloys, etc.).

## Figures and Tables

**Table 1:** Chemical composition of red mud (wt.%).

Fe_2_O_3_	Al_2_O_3_	SiO_2_	CaO	Na_2_O	TiO_2_	Loss
29.10	18.40	17.20	15.90	11.30	5.53	2.57

**Table 2:** The specific composition of the solution.

Component	Concentration (g·L^−1^)	Function
NiSO_4_·6H_2_O	377	Coating
NaBH_4_	2	Reducing agent
NaOH	30	Alkaline environment
C_6_H_5_Na_3_O_7_	50	Buffer

**Table 3:** Chemical element content measured by EDS (at %).

	Area	1	2
Element			
O		53.52	52.68
Fe		12.76	10.57
Al		8.35	11.7
Ni		7.50	9.65
Ca		5.83	2.77
Na		5.28	3.44
Si		5.02	6.3
Ti		1.74	2.89

**Table 4:** EDS analysis of phases at specified locations (at %).

Area	Al	Si	Cu	Ni	Mg	Fe	Suggestion Phase
1	2.28	97.41	0.31				Primary Si
2	39.34	60.42	0.24				Eutectic Si
3	99.15	0.85					α-Al Matrix
4	67.34	2.32	29.93	0.41			θ’-Al_2_Cu
5	63.50	17.13	4.34	0.27	14.76		Q-Al_5_Cu_2_Mg_8_Si_6_
6	68.21		15.14	16.13		0.53	δ-Al_3_CuNi
7	76.18	1.25	16.24	6.33			γ-Al_7_Cu_4_Ni

**Table 5:** Tensile property data of the heat-treated red mud/ZL109 alloys.

Alloys	Content of Red Mud/%	Temperature/°C	UTS (σ_b_)/MPa	YS (σ_s_)/MPa	FS/%	EI/%
1	0	25	265.8	215.3	6.87	5.32
		250	199.6	179.6	3.59	2.11
		300	143.7	120.5	4.37	1.69
		350	94.6	91.3	10.69	2.69
		400	50.0	43.9	10.50	1.99
2	0.5	25	275.2	213.6	6.53	4.98
		250	240.9	226.6	2.78	1.77
		300	149.8	88.2	3.94	1.56
		350	135.7	112.2	4.08	1.99
		400	56.7	49.5	5.50	2.03
3	1	25	295.4	275.8	7.08	6.40
		250	267.8	232.9	3.11	2.16
		300	156.7	123.7	4.51	2.13
		350	143.3	122.9	5.95	1.98
		400	69.0	59.9	10.90	2.41
4	1.5	25	231.7	206.5	5.21	3.75
		250	170.3	144.0	2.47	1.71
		300	155.6	108.7	2.18	1.80
		350	97.8	87.7	7.04	2.07
		400	86.2	80.3	6.40	1.34
5	2	25	216.5	197.4	3.66	2.97
		250	150.4	121.5	2.39	1.98
		300	139.8	99.5	3.905	1.60
		350	95.4	84.3	5.97	1.25
		400	79.0	70.1	6.00	0.98

## Data Availability

The original contributions presented in this study are included in the article. Further inquiries can be directed to the corresponding author.

## References

[1] Javidani M., Larouche D. (2014). Application of cast Al–Si alloys in internal combustion engine components. Int. Mater. Rev..

[2] Hu X., Jiang F., Ai F., Yan H. (2012). Effects of rare earth Er additions on microstructure development and mechanical properties of die-cast ADC12 aluminum alloy. J. Alloys Compd..

[3] Lee C.D. (2008). Variability in the tensile properties of squeeze-cast Al–Si–Cu–Mg alloy. Mater. Sci. Eng. A.

[4] Wazeer A., Das A., Abeykoon C., Sinha A., Karmakar A. (2023). Composites for electric vehicles and automotive sector: A review. Green Energy Intell. Transp..

[5] Cui X., Cui H., Zhao X., Liu F., Du Z., Liang S., Bai P. (2023). Influence of heat treatment on microorganization and mechanical properties and corrosion resistance of Al-Si-Mg-3% Cr alloy. Rare Met. Mater. Eng..

[6] Abdelaziz M.H., Elgallad E.M., Doty H.W., Samuel F.H. (2021). Strengthening precipitates and mechanical performance of Al–Si–Cu–Mg cast alloys containing transition elements. Mater. Sci. Eng. A.

[7] Yang Y., Yu K., Li Y., Zhao D., Liu X. (2012). Evolution of nickel-rich phases in Al–Si–Cu–Ni–Mg piston alloys with different Cu additions. Mater. Des..

[8] Hou L., Peng Y., Zhou L., Zhang G., Xu Y., Gaoming D. (2013). High power density Diesel Engine Aluminum Ppiston Material and Casting Technology. Veh. Engine..

[9] Amanisha A., Wu Y., Li W., Meng F., Wu Y., Liu X. (2022). Al-Ti-C-B crystal alloy on the organization and mechanical properties of Al-12Si-4 Cu-2Ni-1Mg alloy. Precis. Form. Eng..

[10] Li X., Qian W., Sun K., Ge S., Huo C., Wang J., Wang Y. (2023). Al-Si-Cu-Ni-Mg Alloy solidification tissue regulation and influence on high temperature tensile properties. Casting.

[11] Awe S.A., Seifeddine S., Jarfors A.E., Lee Y.C., Dahle A.K. (2017). Development of new Al-Cu-Si alloys for high temperature performance. Adv. Mater. Lett..

[12] Shaha S.K., Czerwinski F., Kasprzak W., Friedman J., Chen D.L. (2016). Ageing characteristics and high-temperature tensile properties of Al–Si–Cu–Mg alloys with micro-additions of Cr, Ti, V and Zr. Mater. Sci. Eng. A.

[13] Rana R.S., Purohit R., Das S. (2012). Reviews on the influences of alloying elements on the microstructure and mechanical properties of aluminum alloys and aluminum alloy composites. Int. J. Sci. Res. Publ..

[14] Bian Y., Gao T., Liu G., Ma X., Ren Y., Liu X. (2020). Design of an in–situ multi–scale particles reinforced (Al_2_O_3_+ZrB_2_+AlN)/Al composite with high strength, elasticity modulus and thermal stability. Mater. Sci. Eng. A.

[15] Xia H., Li M., Zhang G., Huang H., Shi Y., Cai B., Liu Z., Wang J. (2022). Effect of In-Situ TiB2 Particles on the Creep Properties of 3 Wt.% TiB2/Al-Cu-Mg-Ag Composite. JOM-US.

[16] Zhao B., Yang Q., Wu L., Li X., Wang M., Wang H. (2019). Effects of nanosized particles on microstructure and mechanical properties of an aged in-situ TiB2/Al-Cu-Li composite. Mater. Sci. Eng. A.

[17] Li G., Hideo N., Tetsuya H., Dai J. (2000). Improvement of liquid aluminum wetting on Al_2_O_3_. Mater. Eng..

[18] Muñoz M.C., Gallego S., Beltrán J.I., Cerdá J. (2006). Adhesion at metal–ZrO_2_ interfaces. Surf. Sci. Rep..

[19] Ru J., He H., Jiang Y., Zhou R., Hua Y. (2019). Wettability and interaction mechanism for Ni-modified ZTA particles reinforced iron matrix composites. J. Alloys Compd..

[20] Ru J., He H., Jiang Y., Zhou R., Hua Y. (2019). Fabrication and interaction mechanism of Ni-encapsulated ZrO_2_-toughened Al_2_O_3_ powders reinforced high manganese steel composites. Adv. Powder Technol..

[21] Olgun U., Gülfen M., Göçmez H., Tuncer M. (2017). Synthesis and room temperature coating of nano ZrB2 on copper using mechanical roll-milling. Adv. Powder Technol..

[22] Fan L., Wang Q., Yang P., Chen H., Hong H., Zhang W., Ren J. (2018). Preparation of nickel coating on ZTA particles by electroless plating. Ceram. Int..

[23] Guo L., Xiao L.R., Zhao X.J., Song Y.F., Cai Z.Y., Wang H.J., Liu C.B. (2017). Preparation of WC/Co composite powders by electroless plating. Ceram. Int..

[24] Liu X., Han Y., He F., Gao P., Yuan S. (2021). Characteristic, hazard and iron recovery technology of red mud—A critical review. J. Hazard. Mater..

[25] Khanna R., Konyukhov Y., Zinoveev D., Jayasankar K., Burmistrov I., Kravchenko M., Mukherjee P.S. (2022). Red Mud as a Secondary Resource of Low-Grade Iron: A Global Perspective. Sustainability.

[26] Yuan S., Liu X., Gao P., Han Y. (2020). A semi-industrial experiment of suspension magnetization roasting technology for separation of iron minerals from red mud. J. Hazard. Mater..

[27] Zhou G., Wang Y., Qi T., Zhou Q., Liu G., Peng Z., Li X. (2023). Toward sustainable green alumina production: A critical review on process discharge reduction from gibbsitic bauxite and large-scale applications of red mud. J. Environ. Chem. Eng..

[28] Li Y., Zhang H., Han Y., Liu X., Yuan S., Gao P. (2021). Research Progress in red mud recycling. Met. Mine.

[29] Borra C.R., Blanpain B., Pontikes Y., Binnemans K., Van Gerven T. (2016). Recovery of Rare Earths and Other Valuable Metals From Bauxite Residue (Red Mud): A Review. J. Sustain. Metall..

[30] Xu Q., Lin H., Liu H. (2022). Effect of Coating Thickness on Wetting Behavior of Nickel-Coated Alumina by Molten Aluminum. J. Mater. Eng. Perform..

[31] Sui Y., Wang Q., Wang G., Liu T. (2015). Effects of Sr content on the microstructure and mechanical properties of cast Al–12Si–4Cu–2Ni–0.8Mg alloys. J. Alloys Compd..

[32] Asghar Z., Requena G., Boller E. (2011). Three-dimensional rigid multiphase networks providing high-temperature strength to cast AlSi10Cu5Ni1-2 piston alloys. Acta Mater..

[33] Liu H., Pang J., Wang M., Li S., Zhang Z. (2018). High-Cycle Fatigue Behavior and Damage Mechanism of Multiphase Al–Si Piston Alloy at Room and Elevated Temperatures. Adv. Eng. Mater..

[34] Cayron C., Sagalowicz L., Beffort O., Buffat P.A. (1999). Structural phase transition in Al-Cu-Mg-Si alloys by transmission electron microscopy study on an Al-4 wt.% Cu-1 wt.% Mg-Ag alloy reinforced by SiC particles. Philos. Mag. A.

[35] Feng Y., Chen X., Hao Y., Chen B. (2023). Ageing evolution process of theθ ′ -phase in Al-Si-Cu-Mg alloys: Atomic-scale observations and first-principles calculations. J. Alloys Compd..

[36] Biswas A., Siegel D.J., Seidman D.N. (2014). Compositional evolution of Q-phase precipitates in an aluminum alloy. Acta Mater..

[37] Kim K., Bobel A., Brajuskovic V., Zhou B., Walker M., Olson G.B., Wolverton C. (2018). Energetics of native defects, solute partitioning, and interfacial energy of Q precipitate in Al-Cu-Mg-Si alloys. Acta Mater..

[38] Liu H.Q., Pang J.C., Wang M., Li S.X., Zhang Z.F. (2021). Effect of temperature on the mechanical properties of Al–Si–Cu–Mg–Ni–Ce alloy. Mater. Sci. Eng. A.

[39] Lü S., Li S., Yan Z., Wu S., Li J., Ji X. (2024). Preparation of a novel ultra-high strength Al–Si–Cu–Ni alloy at room/elevated temperature by squeeze casting combined with a new heat treatment process. Prog. Nat. Sci. Mater. Int..

[40] Chen H., Wu S., Li J., Zhao D., Lü S. (2024). Effects of Low Nickel Content on Microstructure and High-Temperature Mechanical Properties of Al-7Si-1.5Cu-0.4Mg Aluminum Alloy. Metals.

[41] Zhang Q., Zuo Z., Liu J. (2013). High-temperature low-cycle fatigue behaviour of a cast Al–12Si–CuNiMg alloy. Fatigue Fract. Eng. M.

[42] Sui Y., Wang Q., Liu T., Ye B., Jiang H., Ding W. (2015). Influence of Gd content on microstructure and mechanical properties of cast Al–12Si–4Cu–2Ni–0. 8Mg alloys, J. Alloys Compd..

[43] Zuo L., Ye B., Feng J., Xu X., Kong X., Jiang H. (2019). Effect of δ-Al3CuNi phase and thermal exposure on microstructure and mechanical properties of Al-Si-Cu-Ni alloys. J. Alloys Compd..

[44] Hu K., Xu Q., Ma X., Sun Q., Gao T., Liu X. (2019). A novel heat-resistant Al–Si–Cu–Ni–Mg base material synergistically strengthened by Ni-rich intermetallics and nano-AlNp microskeletons. J. Mater. Sci. Technol..

[45] Qian Z., Liu X., Zhao D., Zhang G. (2008). Effects of trace Mn addition on the elevated temperature tensile strength and microstructure of a low-iron Al–Si piston alloy. Mater. Lett..

[46] Li G., Liao H., Suo X., Tang Y., Dixit U.S., Petrov P. (2018). Cr-induced morphology change of primary Mn-rich phase in Al-Si-Cu-Mn heat resistant aluminum alloys and its contribution to high temperature strength. Mater. Sci. Eng. A.

[47] Pan L., Zhang S., Yang Y., Gupta N., Yang C., Zhao Y., Hu Z. (2020). High-Temperature Mechanical Properties of Aluminum Alloy Matrix Composites Reinforced with Zr and Ni Trialumnides Synthesized by In Situ Reaction. Metall. Mater. Trans. A Phys. Metall. Mater. Sci..

[48] Fu X., Yang H., Wang H., Huang C., Chen Y., Huang Q., Li A., Pan L. (2024). Effect of Mn/Ag Ratio on Microstructure and Mechanical Properties of Heat-Resistant Al-Cu Alloys. Materials.

[49] Zan Y.N., Zhang Q., Zhou Y.T., Liu Z.Y., Wang Q.Z., Wang D., Xiao B.L., Ren W.C., Ma Z.Y. (2020). Introducing graphene (reduced graphene oxide) into Al matrix composites for enhanced high-temperature strength. Compos. Part B Eng..

[50] Mohamed A.M.A., Samuel F.H., Kahtani S.A. (2013). Microstructure, tensile properties and fracture behavior of high temperature Al–Si–Mg–Cu cast alloys. Mater. Sci. Eng. A.

[51] Tang P., He X., Wang X., Li P. (2013). Microscopic organization and Mechanical Properties of Rapid solidification/Powder metallurgy Al-20Si-7.5Ni-3Cu-1Mg-0.25Fe Alloys. J. Aer. Mater..

[52] Meng F., Wu Y., Li W., Hu K., Zhao K., Yang H., Gao T., Sun Y., Liu X. (2022). Influence of the evolution of heat-resistant phases on elevated-temperature strengthening mechanism and deformation behavior in Al–Si multicomponent alloys. Curr. Appl. Phys..

[53] Bugelnig K., Germann H., Steffens T., Sket F., Adrien J., Maire E., Boller E., Requena G. (2018). Revealing the Effect of Local Connectivity of Rigid Phases during Deformation at High Temperature of Cast AlSi12Cu4Ni(2,3)Mg Alloys. Materials.

[54] Belov N.A., Eskin D.G., Avxentieva N.N. (2005). Constituent phase diagrams of the Al–Cu–Fe–Mg–Ni–Si system and their application to the analysis of aluminium piston alloys. Acta Mater..

[55] Sui Y. (2020). Microstructure and High Temperature Properties of Al-Si-Cu-Ni-Mg Cast Heat-Resistant Aluminum Alloy.

[56] Silva E.P., Marques F., Nossa T.S., Alfaro U., Pinto H.C. (2018). Impact of Ce-base mischmetal on the microstructure and mechanical behavior of ZK60 magnesium casting alloys. Mater. Sci. Eng. A.

[57] Yu Y.N. (2020). Principles of metallography.

[58] Fan M., Cui Y., Zhang Y., Wei X., Cao X., Liaw P.K., Yang Y., Zhang Z. (2023). Achieving high strength-ductility synergy in a Mg97Y1Zn1Ho1 alloy via a nano-spaced long-period stacking-ordered phase. J. Magnes. Alloys.

[59] Li Y., Yang Y., Wu Y., Wang L., Liu X. (2010). Quantitative comparison of three Ni-containing phases to the elevated-temperature properties of Al–Si piston alloys. Mater. Sci. Eng. A.

[60] Zhu R., Chen W., Li X., Chen Z., Sui Y., Qu Y. (2024). Effect of Ni on microstructure and mechanical properties of Al-Cu-Mn alloy. Mater. Today Commun..

